# Influence of Binder Composition and Material Extrusion (MEX) Parameters on the 3D Printing of Highly Filled Copper Feedstocks

**DOI:** 10.3390/polym14224962

**Published:** 2022-11-16

**Authors:** Mahrukh Sadaf, Santiago Cano, Joamin Gonzalez-Gutierrez, Mario Bragaglia, Stephan Schuschnigg, Christian Kukla, Clemens Holzer, Lilla Vály, Michael Kitzmantel, Francesca Nanni

**Affiliations:** 1 Department of Enterprise Engineering “Mario Lucertini”, INSTM RU Roma-Tor Vergata, University of Rome “Tor Vergata”, Via del Politecnico 1, 00133 Rome, Italy; 2 Laboratory of Experimental Mechanics, Faculty for Mechanical Engineering, University of Ljubljana, Aškerčeva Ulica 6, 1000 Ljubljana, Slovenia; 3 Institute of Polymer Processing, Montanuniversitaet Leoben, Otto Gloeckel-Straße 2, 8700 Leoben, Austria; 4 Functional Polymers Research Unit, Material Research and Technology Department, Luxembourg Institute of Science and Technology, 5 Rue Bommel, L-4940 Hautcharage, Luxembourg; 5 Industrial Liaison Department, Montanuniversitaet Leoben, Peter-Tunnerstraße 27, 8700 Leoben, Austria; 6 Austrian Research Center, RHP-Technology GmbH, 2444 Seibersdorf, Austria

**Keywords:** additive manufacturing, copper, metals, material extrusion (MEX)

## Abstract

This work aims to better understand the type of thermoplastic binders required to produce highly loaded copper filaments that can be successfully printed via low-cost filament-based material extrusion (MEX). Compounding feedstock material with 55 vol.% of copper and three multi-component binder systems has been performed. The MEX behavior of these feedstocks was evaluated by depositing material at different speeds and appropriately selecting the extrusion temperature depending on the binder composition. The rest of the MEX parameters remained constant to evaluate the printing quality for the different feedstocks. Printable filaments were produced with low ovality and good surface quality. The filaments showed good dispersion of the powder and polymeric binder system in SEM analysis. The feedstock mechanical properties, i.e., the tensile strength of the filament, were sufficient to ensure proper feeding in the MEX machine. The viscosity of the feedstock systems at the adjusted printing temperatures lies in the range of 10^2^–10^3^ Pa·s at the shear rate of 100–1000 s^−1^, which appears to be sufficient to guarantee the correct flowability and continuous extrusion. The tensile properties vary greatly (e.g., ultimate tensile strength 3–9.8 MPa, elongation at break 1.5–40.5%), and the most fragile filament could not be reliably printed at higher speeds. Micrographs of the cross-section of printed parts revealed that as the printing speed increased, the porosity was minimized because the volumetric flow of the feedstock material increased, which can help to fill pores. This study offers new insights into the feedstock requirements needed to produce low-cost intricate copper components of high quality in a reliable and efficient manner. Such components can find many applications in the electronics, biomedical, and many other industries.

## 1. Introduction

Additive manufacturing (AM), also referred to as 3D printing, is defined as the process of joining materials, usually layer by layer, to create objects from 3D model data, as opposed to conventional manufacturing methodologies [1]. AM has gained enormous attention and is transitioning from research to industrial applications due to its ability to produce intricate geometric features that are infeasible or challenging to manufacture by conventional techniques [2]. Another advantage is that no special tooling is required for the production of new geometries with AM technologies; thus, AM is perfect for models fabricated in small production runs [3]. The commercial metal AM techniques are powder bed fusion (PBF) techniques like direct metal laser sintering (DMLS^TM^), selective laser melting (SLM^TM^), electron beam melting (EBM^TM^), and indirect, multi-step processes like vat photopolymerization (VPP), and binder jetting (BJT) combined with sintering [4,5]. The equipment used in direct techniques tend to be more expensive. On the other hand, material extrusion additive manufacturing (MEX) is the most commonly used AM technique for polymers and thermoplastic composites because of its low cost and simplicity [6,7,8,9,10]. Compared with other AM processes, MEX requires a modest initial investment, and it has low material waste, an uncomplicated operating procedure, and reasonable control over processing parameters [6,11,12,13,14]. The advantages of MEX can motivate its implementation and extension to fabricate complex metallic structures with advanced functionality, especially pure copper, which is challenging to process by other commonly used AM techniques like SLM due to high electrical and thermal conductivity [15] that hinder laser sintering. EBM is quite successful in producing highly dense copper components but still has limitations [16]. Moreover, the beam-based AM equipment is expensive, requires high power, and needs special handling of metal powders; and its manufactured components exhibit defects like porosity, cracks, and internal stresses if the process parameters are not optimized [17]. Studies have demonstrated that steel [18,19,20,21], titanium [22,23,24], other metals [25,26,27,28], and also ceramics [29,30] can be processed via MEX. A thermoplastic binder is mixed with sinterable powder to develop a feedstock to achieve metal or ceramic parts through MEX [10]. The feedstock prepared for MEX is comparable to metal injection molding (MIM) feedstocks [5]. The thermoplastic binder is extracted from the MEX-fabricated specimens during debinding. Then, the sintering is performed at temperatures below the melting temperature of the powder to obtain fully densified solid specimens [31].

In most available MEX 3D printers, the material to be deposited is in the form of filaments. The filament is introduced into a liquefier and heated above the binder’s melting temperature. The cooler section of the filament behaves like a piston, pushing the heated fraction and making it flow through the nozzle [32]. Using filaments limits the range of possible materials that MEX can process. The filament should be flexible to be spooled and stiff to avert buckling [10,33]. Nevertheless, challenges with highly loaded filaments can be addressed using an appropriate binder system. This study compares three different binder systems, and their printability is assessed.

In general, the binder system is composed of various types of thermoplastic polymers: (i) backbone to provide structural strength to the printed parts, (ii) additives to provide flexibility to the filament, (iii) waxes to get an optimized feedstock flow and sufficient filament stiffness, and (iv) other additives to enhance powder dispersion and binder adhesion to the powder [10,34,35,36]. Waxes and low molecular weight additives can be used up to a specified amount. Otherwise, the mechanical properties of the filaments and final printed specimen can be severely diminished [36,37], thus requiring extreme care during handling to prevent breakage.

Few studies on the 3D printing of copper by filament-based MEX are available [38,39]. They used commercially available highly filled copper filaments to print copper components to study printability using different machines, binders, and powders, so it is difficult to draw a solid conclusion on the different printability behaviors. In our study, the same copper powder is mixed with three multi-component binder systems and printed under similar conditions in the same MEX machine. The effect of the binder system on the 3D printing performance is compared and evaluated for all feedstocks.

This study aims to comprehensively understand the type of binder needed to make filaments loaded with copper that can be successfully printed using a low-cost MEX machine. The preparation of feedstocks with 55 vol.% copper powder and three unique multi-component binder systems was performed. Three different polyolefins were selected as backbone components because of their excellent viscosity and low cost [40]. These polymers decompose with small volumetric changes; thus, diminishing the probability of failures during debinding and sintering. Further additives like styrene-ethylene-butylene copolymer (SEBS), paraffin wax (PW), and stearic acid (SA) were also used to achieve stiffness, flexibility, and appropriate viscosity. The filaments were extruded with the different feedstocks, and morphological and mechanical characterization was performed. Filament characterization included scanning electron microscopy (SEM) and optical microscopy to observe the dispersion of copper powder in the binders. Tensile tests were conducted to determine the mechanical properties of the filaments, and a high-pressure capillary rheometer was used to measure the viscosity of feedstocks. The influence of printing parameters on the quality of MEX specimens produced with the three feedstocks was also studied, including the effect of the printing speed, printing temperature, and extrusion speed.

## 2. Materials and Methods

### 2.1. Materials and Preliminary Characterizations

Copper powder with a purity of 99.9% (Cu 99.9, Carpenter Powder Products Inc., Woonsocket, RI, USA) was used in this study. The particle size distribution details of the powder provided by the supplier are shown in Table 1. The phase analysis of copper powder was investigated by X-ray diffraction (XRD, Philips X’Pert 1710, Bragg-Brentano, Netherlands). XRD patterns were recorded in the 2θ range 10–90° in the following conditions: Cu Kα radiation (λ = 1.5408 Å), 40 kV and 40 mA, step size = 0.020°, time per step = 2 s.

Two multicomponent binder systems developed at the Montanuniversitaet Leoben (i.e., B1_ST, and B2_ST) and a third composition at the University of Rome Tor Vergata (B3_T) were used in this research. Binder B1 was previously used for specimen fabrication of 316-L steel [41], 17-4PH steel [42], hard-metal [27], cermet [27], zirconia [29], alumina [43], copper [16], titanium [22] and nickel-base superalloy [28]. B1_ST contains a grafted polyolefin (BYK Chemie GmbH, Wesel, Germany), but the rest of the formulation is confidential. Binder B2_ ST was used to fabricate zirconia specimens [44] and it is composed of 35.8 vol.% commercial acrylic-acid grafted high-density polyethylene (AAHDPE) (SCONA TPPE 2400, BYK-Chemie GmbH, Wesel, Germany) having a density of 0.94 g/cm^3^. As reported by the supplier [45], the material contains a minimum of 5 wt% of acrylic acid and has a melt volume rate (MVR) ranging from 9 to 20 cm^3^/10 min (190 °C, 2.16 kg). The rest of the composition includes 27 vol.% of styrene-ethylene-butylene copolymer (SEBS) (MD1653, Kraton Polymers Nederland B.V., Amsterdam, Netherlands) having a density of 0.9 g/cm^3^, a melt flow rate of 25 g/10 min (230 °C, 2.16 kg), and a styrene/rubber ratio of 30/70; 27 vol.% of paraffin wax (Sasolwax 6403, Sasol Wax GmbH, Hamburg, Germany) having a density of 0.9 g/cm^3^; and 10.2 vol.% of stearic acid (Merck Schuchardt OHG, Hohenbrunn, Germany) having a density of 0.941 g/cm^3^. Binder B3_T was used to produce 316L stainless steel specimens [21], and it is composed of 97 vol.% of low-density polyethylene (LDPE RIBLENE MV 10 R ENI Versalis S.p.A, Milan, Italy) as the main backbone. According to the supplier [46], the MVR of LDPE is 16 cm^3^/10 min (190 °C, 2.16 kg) and the density 0.91 g/cm^3^. The other ingredient in B3_T is 3 vol.% of stearic acid (Merck Schuchardt OHG, Hohenbrunn, Germany) as a surfactant having a density of 0.941 g/cm^3^. Binders B1_ST and B2_ST need solvent (S) and thermal (T) debinding, while binder B3_T needs only thermal debinding; thus, the letters S and T after the underscore in their abbreviated names.

### 2.2. Compounding of Binders

All binders were prepared in a co-rotating twin screw-extruder ZSK 25 (Werner & Pfleiderer GmbH, Dinkelsbühl, Germany) as described in [29]. The binder ingredients were premixed in solid state before melt compounding. Compounding temperatures ranged from 25 to 170 °C from hopper until the extrusion die; the rotational screw speed was 150 rpm and the feeding rate was 7 kg/h. All processing temperatures had to be adjusted based on the binder composition because binder components had different melting temperatures. A water bath was used to cool down the extrudate, an air blade was used to remove the surface moisture, and finally a strand pelletizer (SGS 50-EL, Scheer Reduction Engineering GmbH, Wesel, Germany) was used for granulation. The obtained pellets were dried in a vacuum drying oven FDL 115 (Binder GmbH, Bottrop, Germany) at 40 °C for 12 h before proceeding to compound the feedstocks. Seven kilograms of each binder system were prepared.

### 2.3. Compounding of Feedstocks

The feedstocks were prepared by compounding 55 vol.% copper powder and the three binder systems described above. A Leistritz ZSE 18 HP-48D co-rotating twin screw-extruder (Leistritz Extrusionstechnik GmbH, Nuremberg, Germany) was used. Two gravimetric feeding units were used to convey binder pellets and the metal powder into the extruder. A rotational speed of 600 rpm and a feeding rate of 20 kg/h were used in all compounding trials. The extrusion parameters are detailed in Table 2. Please note that, the compounding temperature is the temperature set in zones 1 to 11 of the compounder and were adjusted based on the binder composition. The compounded feedstock was collected using a conveyor belt, where it cooled down by natural convection. The cooled feedstock was granulated with a Retsch SM200 cutting mill (Retsch GmbH, Haan, Germany) fitted with a sieve having 4 mm × 4 mm square openings. The obtained pellets had a length between 2 and 3 mm.

### 2.4. Binder and Feedstocks Filament Extrusion

The mechanical properties of feedstock filaments are influenced by the mechanical properties of their binder. For this reason, binder filaments with a proper diameter and low ovality were extruded and mechanically tested. An FT-E20T-MP-IS single screw extruder (Dr. Collin GmbH, Maitenbeth, Germany) with three heating zones, and a die diameter of 1.75 mm was selected to prepare the filaments. The extrusion line had a conveyor belt to pull the extruded filaments followed by a haul-off unit, and a Diagnostic Laser 2000 (SIKORA AG, Bremen, Germany) to continually measure the diameter and ovality of the produced filaments. Finally, an automatic spooling device was used to continuously spool the filaments. The extrusion temperature, screw rotational speed, and haul-off unit were adjusted for each binder to ensure an appropriate filament diameter. The extrusion processing parameters for binders and feedstocks are summarized in Table 3.

Feedstock filaments were also extruded using the same equipment as binder filaments. Filaments were manufactured by using the three previously compounded and pelletized feedstocks. The extrusion temperature and speed were tuned for each feedstock according to the binder composition. The behavior during processing varied depending on the binder composition. Filaments containing B1_ ST-based feedstock (F1_ST) could be spooled automatically. However, filaments from F2_ ST and F3_T feedstocks did not have sufficient strength to withstand the forces exerted by the spooling unit during the automatic spooling process; thus, spooling was done manually. Nevertheless, it was possible to measure their diameter and ovality during manual spooling.

### 2.5. Thermal Properties of the Feedstocks: Differential Scanning Calorimetry (DSC)

The melting and crystallization temperature of the binders and feedstocks were measured with differential scanning calorimetry (DSC). The measurements were conducted under a protective nitrogen atmosphere using a Mettler Toledo DSC 1 equipped with a gas controller GC 200 (Mettler Toledo GmbH, Greifensee, Switzerland). The temperature program was as follows: heating rate set to 10 K min^−1^ from 30 to 260 °C; cooling rate set to −10 K min^−1^ from 260 to 30 °C. Three repetitions were performed for each material.

### 2.6. Rheological Analysis of Binders and Feedstocks

The binder and feedstock rheological properties were analyzed with a Rheograph 2002 high-pressure capillary rheometer (Goettfert Werkstoff-Prufmaschinen GmbH, Buchen, Germany). Viscosities were measured at apparent shear rates between 100 and 1000 s^−1^. Different testing temperatures were selected for each composition according to the results of the preliminary printing trials with the feedstocks. For binder B1_ST and feedstock F1_ST, a temperature of 255 °C was used. Viscosities for binder B2_ST and feedstock F2_ ST were measured at 200 °C, while for binder B3_T and feedstock F3_T at 180 °C. The capillary rheometer was fitted with round dies with a 1 mm diameter, and three different lengths (10, 20, and 30 mm). A pressure sensor capable of measuring up to 1000 bars was used for the die with a length of 30 mm. Another sensor with a maximum pressure of 500 bars was used for the 10 and 20 mm long dies. The measurements were performed in triplicate to ensure the repeatability of the results. After using the Weisenberg-Rabinowitsch [29,47] and Bagley [48] corrections, the true viscosity and true shear rate values were calculated, respectively.

### 2.7. Morphology of Feedstock Filaments

The feedstock’s morphology was analyzed by SEM (Zeiss SEM-FEG Leo, supra-35, Dresden, Germany) coupled with energy dispersive spectroscopy (EDS) (INCAx-sight, Oxford Instruments, Abingdon, UK). The observations were performed in filament samples that were previously cryo-fractured in liquid nitrogen and gold sputtered (10 nm).

### 2.8. Tensile Testing of Filaments

Tensile tests were conducted on the binder and feedstock filaments. Straight filaments of a diameter of 1.75 mm with a gauge length of 50 mm were tested in a universal testing machine (Lloyd LRX, AMETEK, Inc., Berwyn, IL, USA) under standard conditions (i.e., 23 °C and 50% humidity). Loadcells of 2.5 kN for the binder and 500 N for feedstocks were selected for testing. The tests were performed at 10 mm min^−1^ until the specimen ruptured. LLOYD vice grips were used to fix the filaments, and the deformation was analyzed by ONDIO application software. Five measurements were performed with each material.

### 2.9. Additive Manufacturing by MEX

**Figure 1:**
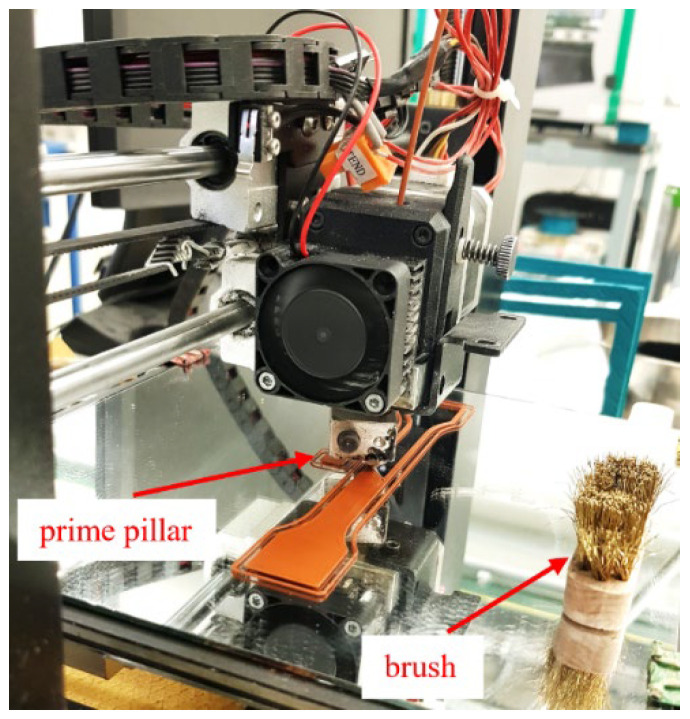
Fabrication of the specimens with a prime pillar and additional brush.

The processability by MEX of the three developed highly filled filaments was investigated. Preliminary printing trials were conducted to determine the extrusion temperature and flow rates for each type of feedstock. Several types of specimens having different geometries (i.e., parallelepipeds, cones, cylinders, and pyramids) were printed. Moreover, bending specimens, according to ISO 178:2001, of 80 mm in length, 10 mm in width, and 4 mm in thickness and tensile test specimens (having dimensions 150 mm × 20 mm × 4 mm according to ISO 527) were produced. 3D printing was conducted on a Duplicator i3 v2 (Wanhao, Jinhua, China) MEX machine. The software Simplify 3D version 4.1.2 (Simplify3D, Blue Ash, OH, USA) was used to prepare the G-code for 3D printing. A TwinClad XT-coated brass nozzle MK10 with a diameter of 0.4 mm was used. The processing parameters that were varied are shown in Table 4. As shown in Table 4, specimens were produced with two printing speeds (i.e., 10 mm s^−1^ and 60 mm s^−1^) to study the influence of the printing speed on the density, production time, and overall quality of the specimens printed with the different feedstocks. A printing platform temperature of 100 °C, infill orientation of ±45°, infill density of 100%, outline-perimeter overlap of 50%, flow multiplier of 110%, speed multiplier of 100%, layer thickness of 0.2 mm, and four perimeter lines were kept constant in all printing trials. A glass mirror was used as the build platform material. A fixative PVP-based spray coating was applied to the glass mirror to ensure proper adhesion of the first layer. To overcome the accumulation of material at the die due to overflow, an extra brushing step and “prime pillar” were added. A brass brush was mounted at the corner of the build platform (Figure 1). The G-codes contained a brushing step after each layer to remove accumulated material from the nozzle during specimen fabrication. A “prime pillar” (Figure 1) was printed near the tensile bar specimens in each batch by selecting this function in the slicing software. First, a layer of the prime pillar was printed to remove the excess material flowing out of the nozzle after the brushing step to prevent material accumulating around the corners and surface of the mechanical testing specimens. The prime pillar also ensured the nozzle was fully filled with material before continuing with the actual specimens. Such a tower structure has been previously used to improve the quality of multi-material specimens [49]. For each material and each printing speed, six specimens were produced in two batches, three specimens at the same time.

### 2.10. Morphological Characterization of Printed Specimens

For each feedstock, morphological analysis of the specimen’s cross-section was performed to study the powder distribution in the polymer binder and identify if any production defects had occurred. Samples were cut at a length of 10 mm from the end of the 3D printed bending specimens with a cutting machine (Discotom, Struers Inc., Cleveland, OH, USA). The cut samples were examined using a VHX 3D Microscope (Keyence Co., Osaka, Japan) at magnifications of 20× and 200×.

## 3. Results and Discussion

### 3.1. Raw Material Characterization

**Figure 2:**
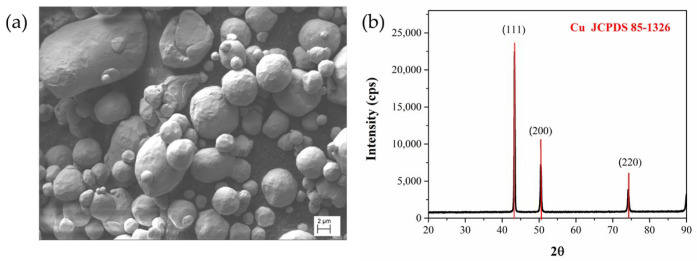
(**a**) The SEM image of the copper powder with magnifying power of 2.29 KX as provided by the supplier; (**b**) XRD pattern of the copper powder in the 2θ range 20–90°.

The morphology of the copper powder as provided by the supplier was analyzed by scanning electron microscopy (SEM) (Figure 2a). Most of the particles were spherical or quasi-spherical. The average particle size is 16 µm according to the datasheet. The chosen powder size is known to offer a high packing density and enhanced flowability [50,51]. A high powder flowability reduces the feedstock viscosity by providing less resistance to flow with the binder, facilitating 3D printing even at high powder contents [23,52]. On the other hand, a high powder packing density means more powder per unit volume of feedstock, which decreases the overall shrinkage during sintering [23]. The chosen particle size has also the advantage of avoiding complications during 3D printing like the obstruction of the MEX machine nozzle since it has an opening diameter between 0.2 and 0.8 mm [10]. Moreover, this copper powder has been sintered successfully after preparing specimens by MEX [16]. The X-ray diffraction pattern of the copper powder is shown in Figure 2b. Sharp peaks can be seen at 2θ = 43.6°, 50.7°, and 74.45°, representing (111), (200) and (220) planes of a face-centered cubic structure, as confirmed by the reference pattern JCPDS 85-1326.

### 3.2. Filament Production, Morphology, and Microstructure of Filaments

As previously explained in Section 2.4, the processing behavior and quality of each of the feedstock filaments was different, depending on the binder composition. During extrusion, the diameter and ovality of the filaments were monitored and the processing parameters were adjusted accordingly, to obtain round filaments having a diameter of 1.75 ± 0.05 mm.

The diameter of the filaments greatly impacts the material printability and quality of the final printed specimens. If the filament diameter is not within tolerances, particularly if it is lower than the set value (i.e., 1.75 ± 0.05 mm), the mass flow rate of deposited material is lowered resulting in uneven thicknesses and widths of the layers, arising in poor interlayer adhesion and/or presence of unwanted voids within or between layers. On the contrary, if the filament is thicker than the set value, the filament feeding to the nozzle may be difficult, and blocking is not uncommon. Extrusion of thicker filaments leads to an overflow of material, which results in poor dimensional accuracy [53,54]. To avoid such challenges, the filament’s diameter was monitored carefully, and the extrusion parameters (i.e., rotational screw speed, haul-off speed, and winding speed) were adjusted to achieve filaments of an average diameter of 1.75 mm. Table 5 shows the diameter and ovality over the entire length of the filaments in the spools used for producing specimens with all three types of feedstocks.

It must be noted that ovality is defined as the difference in the major and minor axes of the describing ellipse. Consequently, a filament with zero ovality is fully round as both axes are equal [19]. All the feedstock filaments had a low ovality (Table 5), confirming that the filaments were sufficiently round. The roundness of the filaments guarantees a suitable grip by the feeding system and prevents slippage during feeding [54]. For the detailed diameter distribution over the length of the spooled filaments prepared during this investigation, please see Figure S1 in the Supplementary Materials.

**Figure 3:**
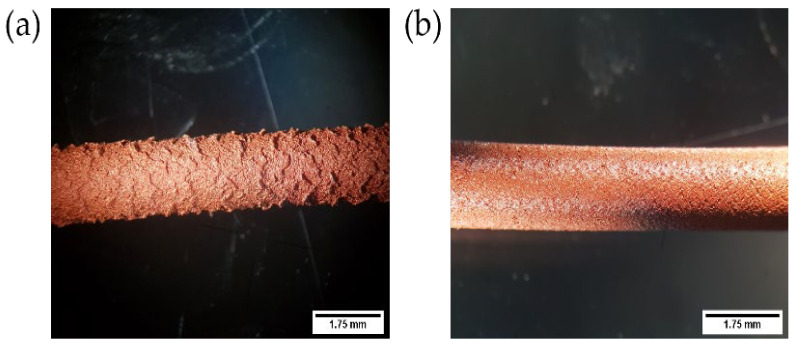
Example of an extruded filament of feedstock based on the B3_T binder system: (**a**) Poor filament surface quality at an extrusion temperature of 160 °C representing “shark skin”; (**b**) improved surface quality at 175 °C.

Figure 3 shows the influence of the extrusion temperature on the surface quality of the filament. Initially, a temperature of 160 °C was set to produce F3_T filaments, but the low temperature led to a rough surface, as shown in Figure 3a. These irregularities on the surface of the extrudate are referred to as “shark skin” or “shark skin melt fracture” [55]. It is directly related to the melt viscosity and thus to the extrusion temperature and extrusion speed (shear stresses). Shark skin is triggered by the high strain rate when the extrudate leaves the extruder die’s opening. As the outer layer of the molten feedstock undergoes stretching and sharp acceleration, rupture happens above a certain critical stress value [55,56,57]. In this case, the temperature played a crucial role since a lower extrusion temperature (i.e., 160 °C) resulted in the high viscosity of the feedstock and finally shark skin was observed.

Increasing the temperature to 175 °C led to a smoother outer surface without visible defects (Figure 3b). As the extrusion temperature increases, the mobility of the polymeric chains increases, which lowers the viscosity of the feedstock [58,59]. Reduction in viscosity prevented defects during extrusion even at higher shear rates (i.e., screw rotational speeds).

**Figure 4:**
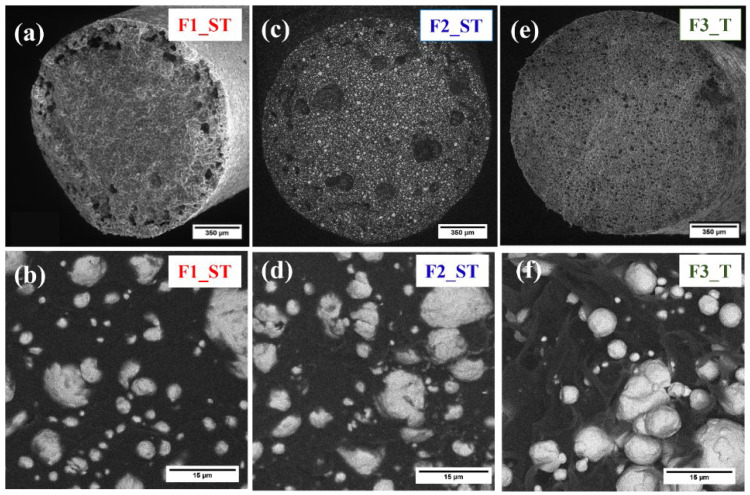
Morphology of feedstock filaments: F1_ST with a magnifying power of (**a**) 150 kx; (**b**) 5 kx: F2_ST with a magnifying power of: (**c**) 150 kx; (**d**) 5 kx: and F3_T with a magnifying power of (**e**) 150 kx; (**f**) 5 kx.

The SEM micrographs of the cross-section of the feedstock filaments F1_ST, F2_ST, and F3_T are shown in Figure 4. Porosity was observed in all three types of feedstock filaments. Notably, the three filaments showed different types of porosity in terms of location and dimension. In particular, the F1_ST filament had most of the porosity in the outer section (Figure 4a). In comparison, the F2_ST filament had larger pores distributed all over the entire cross-section (Figure 4c); finally, in the F3_T filament, small pores were homogeneously distributed throughout the cross-section (Figure 4e). The observed pores most likely were the result of air trapped during the plasticizing step during extrusion. It was observed that the copper powder was well dispersed in the binder system. The individual copper particles were fully covered by the matrix polymer for F2_ST and especially for F1_ST (Figure 4b,d). Such morphology in the filaments is a prerequisite to achieve a homogenous powder distribution in the 3D printed specimens. While in the F3_T filament, the binder did not fully coat the particles and binder fibrils were formed. It was also observed that an inhomogeneous powder distribution in F3_T was obtained (Figure 4f).

### 3.3. DSC Results

**Figure 5:**
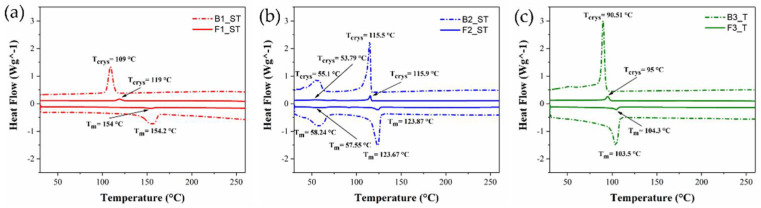
DSC results for the binder system and feedstock at a temperature range of 25–260 °C: (**a**) B1_ST and F1_ST; (**b**) B2_ST and F2_ST; (**c**) B3_T and F3_T.

The thermal properties of the binders and feedstocks were obtained using DSC. Table 6 shows the results obtained by DSC. For the diagram of heat flow versus temperature, please see Figure 5.

As expected, the three-binder compositions show different thermal properties; in particular, the binders B1_ST and B3_T present one melting peak at 153 °C and 103 °C, corresponding to the melting of the backbone polymers, respectively. On the other hand, the binder B2_ST shows two endothermic peaks ascribed to the melting of stearic acid and paraffin wax (primary binder) at 58 °C and backbone polymer (AAHDPE) at 124 °C. The presence of the copper particles in the feedstock does not significantly affect the melting temperatures. On the other hand, the crystallization temperatures of feedstock F1_ST and F3_T increase when the copper particles are present. This result could be related to the high thermal conductivity of the copper particles that may also act as nucleation points during cooling even though they have a micrometrical size. These results, along with those coming from the rheological tests, are key to setting the proper 3D printing parameters. The nozzle temperature should be set higher than the T_m_ of the feedstock material to have constant extrusion. Nozzle temperatures well above the T_m_ improve the polymeric chain mobility and enhance molecular diffusion among the adjacent strands while printing [60]. When using semi-crystalline polymers, including polyolefins, setting the build platform temperature above their glass transition temperature can minimize warpage and detachment from the platform [61]. However, the temperature of the building platform should be lower than the T_crys_. A higher bed temperature helps to enhance interfacial bond strength, as the time before the onset of crystallization is important for molecular diffusion and stress relaxation [60].

### 3.4. Rheological Properties of Binders and Feedstocks

**Figure 6:**
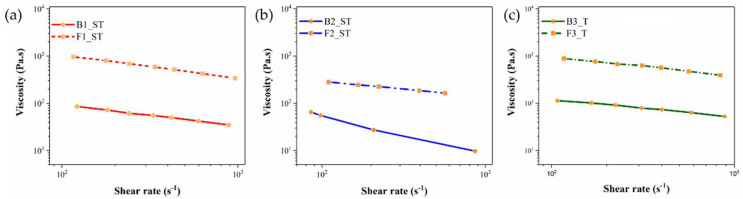
Viscosity as a function of shear rate for the binder system and feedstock system: (**a**) B1_ST and F1_ST at a temperature of 255 °C; (**b**) B2_ST and F2_ST at a temperature of 200 °C; (**c**) B3_T and F3_T at a temperature of 180 °C.

The binder and feedstock viscosities were measured at the best printing temperatures obtained in preliminary trials with the different feedstocks. The temperatures were 255 °C for F1_ST and B1_ST, 200 °C for F2_ST and S2_ST, and 180 °C for F3_T and B3_T. The curves of true viscosity versus the true shear rate are shown in Figure 6. The curves lucidly show that the feedstock viscosities are higher than that of the neat binders. As expected, the viscosity increases after incorporating copper particles into the binder since the produced particle-particle network limits the mobility of the polymeric chains; also the particle-binder interaction and interparticle friction contribute to the increase in viscosity [29,62]. As seen in Figure 6, the viscosity at lower shear rates is higher as the particle-particle network restricts binder flow. However, at certain limits, the network breaks, and the material flows easier. It is essential that a suitable viscosity is achieved in the MEX nozzle to guarantee proper flow of the molten feedstock, so it can be smoothly deposited to the build surface [21]. When comparing the viscosity values of the different systems, it can be observed that F1_ST and F3_T have similar viscosity values in the range of the shear rates investigated, while F2_ST has lower viscosity values (Figure 6). According to the literature, the viscosity of a suitable MEX feedstock should be between 100 and 1000 Pa·s [63,64]. As can be observed in Figure 6, the feedstock viscosity at the selected printing temperatures lies in the correct range at shear rates of 100–1000 s^−1^, which was sufficient to ensure the appropriate and continuous flow during the preliminary printing trials. Care should be taken because for filament-based MEX having the correct viscosity is not sufficient to achieve printable materials; the mechanical properties of the filament also need to be considered (see Section 3.5).

### 3.5. Mechanical Properties of Binders and Feedstocks

**Figure 7:**
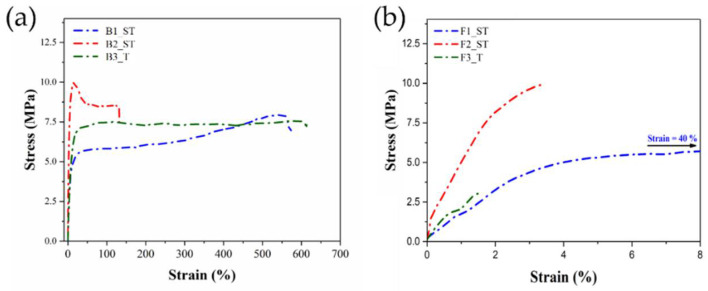
Stress-strain curve of filaments of (**a**) binder system (B1_ST, B2_ST & B3_T); (**b**) feedstock system (F1_ST, F2_ST & F3_T).

The mechanical properties of the filaments have a significant impact on the processability of filaments-based MEX 3D printing. Sufficient flexibility and strength are necessary to spool the filament during extrusion and un-spool it during printing. Furthermore, the filament should have enough stiffness to prevent buckling during printing [65]. As different binders were used in this study, completely different mechanical properties were observed. Representative stress-strain curves of filaments produced with the different binder and feedstocks are shown in Figure 7. Two important parameters obtained from stress-strain curves are the maximum stress and strain at break (Table 7). As indicated in Figure 7a, B1_ST and B3_T binder systems showed a very high elongation at break (546.6 ± 156.9% and 583.75 ± 78.4%) and maximum stresses (7.9 ± 1.4 MPa and 7.6 ± 1.4 MPa). In contrast, B2_ST showed lower elongation at break (16.6 ± 1.4%) and the highest maximum stress (9.9 ± 0.14 MPa). For the three systems studied, the incorporation of the powder resulted in a decrease in the mechanical properties of the filaments, which was different in each case. In Figure 7b, it is shown that the feedstock filament F1_ST has a very high elongation at break (40.5 ± 4.3%) and a maximum stress (i.e., σ_max_ = 7.95 ± 0.12 MPa). In comparison, feedstock filaments F2_ST had the lowest elongation at break (3.3 ± 1.16%) and highest maximum stress values (i.e., σ_max_ = 9.8 ± 0.17 MPa). The feedstock filament F3_T showed the lowest elongation at break (1.5 ± 0.24%) and the lowest maximum stress (i.e., σ_max_ = 3.0 ± 0.12 MPa) of all feedstocks.

The different trends observed in the mechanical properties of binders and feedstocks can be explained by the different interactions of the binder components with the powder in each of the tested formulations. For F1_ST, the high binder flexibility in B1_ST is combined with sufficient adhesion to the copper powder, which is homogeneously dispersed in the polymeric matrix (Figure 4b). The high mechanical properties of the binder combined with the good powder dispersion results in filaments that can be easily processed, as observed in the extrusion trials. On the other hand, the poor powder-binder adhesion in F3_T (Figure 4f) results in a significant reduction in the original filament flexibility in B3_T. In the case of F2_ST, despite having a better powder-binder adhesion than F3_T, the low flexibility of B2_ST also results in fragile filaments that need careful handling, as mentioned in Section 2.4. These results demonstrate that the binder-powder interaction is crucial in determining the mechanical properties of the feedstock filament. The binder-powder interaction changes with the different additives used in the different feedstocks. However, despite F2_ST and F3_T being more fragile than F1_ST, their properties are comparable with other filaments reported [10,66,67]. Thus, these mechanical properties are supposed to guarantee a correct feeding during the MEX process.

### 3.6. Influence of Printing Temperature, Extrusion Multiplier, and Printing Speed on 3D Printed Specimens

#### 3.6.1. Influence of Printing Temperature

DSC measurements were performed (Section 3.3) to select the nozzle temperature by estimating the melting point (T_m_) of the feedstocks. The nozzle temperature has to be significantly higher than the melting temperature of the polymer binder in the feedstock, not only to melt it, but also to decrease the viscosity of the feedstocks for continuous extrusion to occur [41].

**Figure 8:**
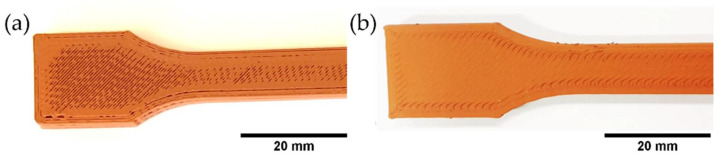
Top views of the fabrication of tensile bar based on F1_T filaments: (**a**) Printing trial at a temperature of 220 °C; (**b**) printing trial at a temperature of 255 °C.

For the feedstock F1_ST, the printing trials were conducted with nozzle temperatures between 220 and 255 °C. At the lowest temperature (i.e., 220 °C), the material did not flow correctly due to the high viscosity. Less material extruded out of the nozzle resulted in printed specimens with visible gaps between the extruded lines (Figure 8a). While at the highest nozzle temperature (i.e., 255 °C), the polymeric chains had a higher mobility and thus lower viscosity, which produced the correct flow of the material. Trials at excessively higher temperatures (>255 °C) could not be performed due to hardware limitations. The nozzle temperature was limited by the heater in the 3D printer and stability of the PTFE guiding tube in the nozzle could be compromised. Nevertheless, an excessively high nozzle temperature has an undesirable effect on dimensional accuracy due to overflow and slow solidification [60]. Correspondingly, polymer degradation can occur if the temperature is too high resulting in reduced mechanical properties [68].

**Figure 9:**
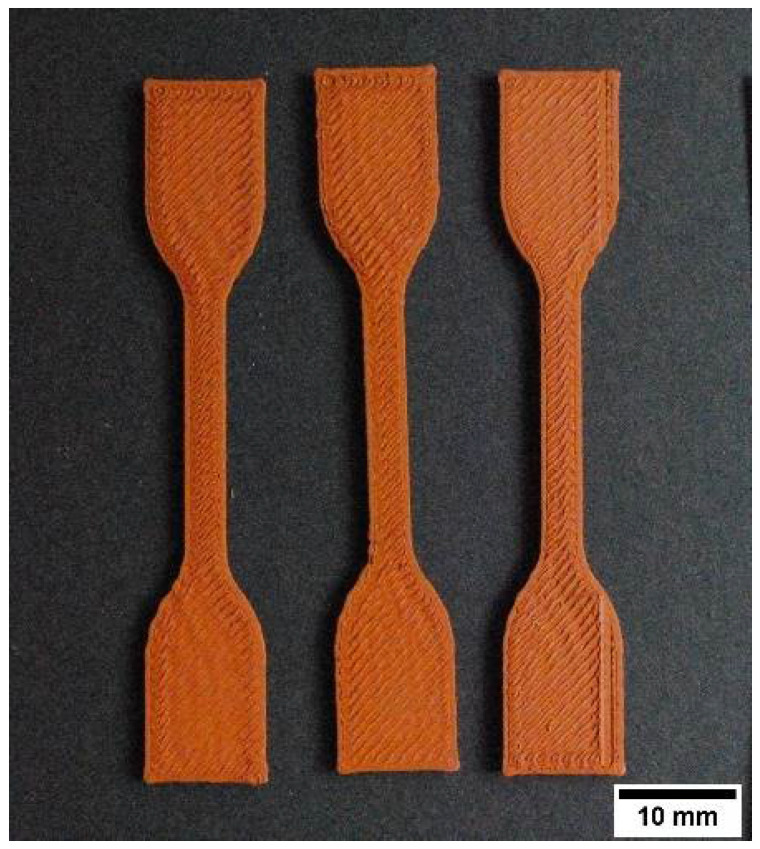
Tensile bar produced with F2_ST filaments at a temperature of 200 °C.

Feedstock F2_ST was 3D printed with a nozzle temperature set between 170 and 210 °C. Printing at 200 °C resulted in printed specimens with good properties (Figure 9). Printing trials at 210 °C had to be stopped because this feedstock contained a high quantity of wax and stearic acid that degraded into volatile gases. Therefore, the printing of F2_ST was limited to a maximum temperature of 200 °C to avoid polymer degradation and the formation of porosity in the printed specimens [29].

**Figure 10:**
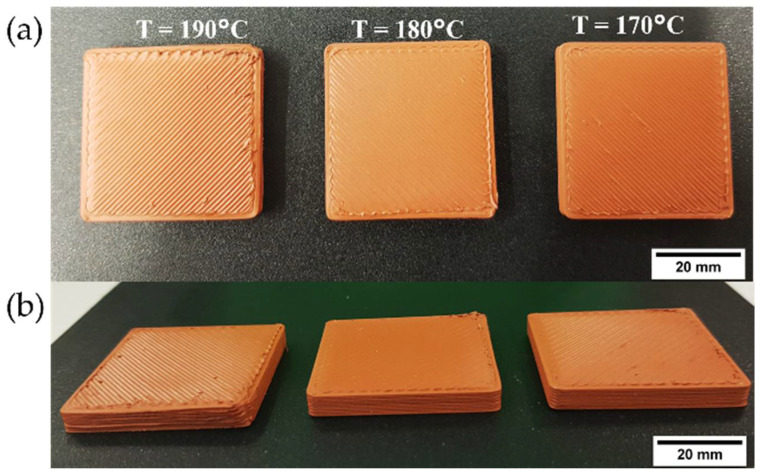
Parallelepiped specimens based on F3_T filaments to study the influence of printing temperature during preliminary printing trials: (**a**) Top view; (**b**) side view, providing a clear view of printed corners.

An example of the parallelepiped specimens produced with F3_T with nozzle temperatures of 170, 180 and 190 °C can be seen in Figure 10. The nozzle temperature of 180 °C resulted in the best results. Higher temperatures (i.e., 190 °C) led to overflow due to very low viscosity resulting in an inhomogeneous extrusion. Furthermore, excess material accumulated around corners and on the top surface around the perimeter lines (Figure 10b). The rough edges were the result of overheating and slow solidification of F3_T. On the other hand, the printing at 170 °C resulted in insufficient extrusion and gaps between the extruded lines (Figure 10).

#### 3.6.2. Influence of Extrusion Multiplier

**Figure 11:**
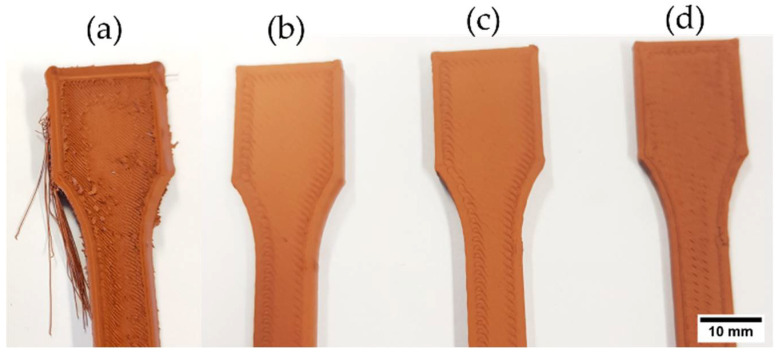
Example of fabrication of tensile specimens based on F2_ST filaments to study the influence of the flow multiplier during preliminary printing trials: (**a**) Extrusion multiplier 130%; (**b**) extrusion multiplier 110%; (**c**) extrusion multiplier 105%; (**d**) extrusion multiplier 100%.

The extrusion multiplier (i.e., flow rate) is another 3D printing parameter significantly influencing material printability, and it has to be chosen carefully based on the material being processed. The extrusion multiplier controls the amount of material extruded through the nozzle. A 100% extrusion multiplier corresponds to the standard flow rate. Extrusion multipliers higher than 100% ensure more material is being extruded per unit time, resulting in fewer and smaller printing voids and a better overlap with the perimeter layer [69]. A low extrusion multiplier value causes insufficient material deposition, forming large internal and surface voids resulting in a visibly poor surface finish. In Figure 11, an example of tensile specimens printed with F2_ST filaments to investigate the influence of variations in a flow multiplier using a 0.4 mm nozzle and an optimized temperature of 200 °C. As expected, having a flow multiplier of 100% resulted in significant sections not being filled properly and visible surface voids. By increasing the flow multiplier up to 110%, specimens with tight dimensional accuracy and good appearance were printed. This result may be related to the porosity observed in the extruded filaments (Figure 4). A volumetric flow rate of 110% compensates for the under-extrusion of material due to the filaments porosity and ensures complete filling resulting in minimal porosity in the printed parts and improved green density, in accordance with previous investigations [10,69]. However, too high a volumetric flow of 130% leads to unsuccessful printing, as clearly shown in Figure 11a. As the volumetric flow increased, the overflow of material results in material accumulation around the nozzle, which then is lost as pieces on top of the parts. Moreover, the overflow results in undesired extrusion when the printing head moves to other areas of the parts, causing the extruded threads outside of the parts. The same phenomena as for F2_ST was observed for F1_ST and F3_T at low and high extrusion multipliers. Therefore, the value of 110% was chosen for the three feedstocks.

#### 3.6.3. Influence of Printing Speed

**Figure 12:**
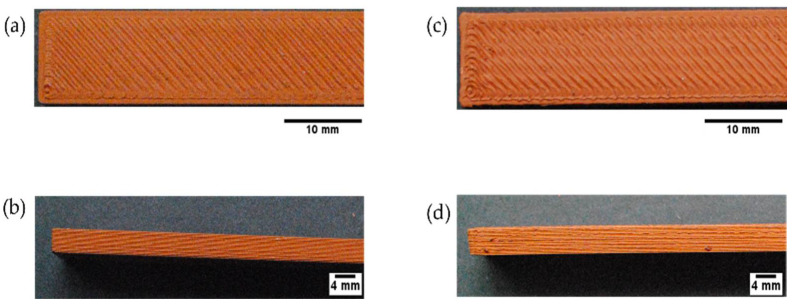
Top and side view of bending specimens based on F1_ST filaments to study the influence of printing speed: (**a**,**b**) Speed 10 mm s^−1^; (**c**,**d**) speed 60 mm s^−1^.

Variations of printing speed were also investigated. The speed of the printing head has a significant impact on the morphology of the extruded material and the appearance of printed specimens [69]. Bending specimens fabricated with F1_ST filaments were used to study the influence of printing speed. The results are shown in Figure 12 and the surface quality obtained with a lower speed (10 mm s^−1^) was much better, compared to a higher speed (60 mm s^−1^). When using a lower speed, the surface of the deposited layers was flatter, and the corners of the parts were sharper. This is because at a lower speed the extruded material shape was more homogenous. Furthermore, as suggested by other studies [69], a higher printing speed increases the volumetric flow through the nozzle. As a result of the increased volumetric flow, the pressure drop across the nozzle increases. This higher pressure drop resulted in deformed edges and the formation of rougher surfaces in the printed specimens.

**Figure 13:**
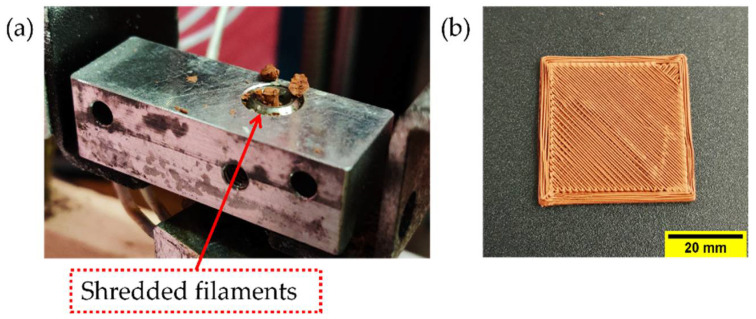
During printing of F3_T feedstock filaments, material shredded in the extruder at a speed of 60 mm s^−1^: (**a**) Hot end; (**b**) inconsistent extrusion due to filament breakage.

When printing F3_T-based filaments at speeds higher than 37.50 mm s^−1^, the filament was constantly breaking between the bottom of the feeding gears and heating block (Figure 13). Because the F3_T filament was very fragile, it could not withstand the compressive forces and pinching of the feeding wheels required to extrude at high speeds. The increase in printing speed causes an increase in the material’s volumetric flow across the nozzle. As previously mentioned, an increase in flow rate increases the pressure drop at the exit of the nozzle; therefore, a higher compressive force is needed to maintain a continuous flow of material [50,70,71]. Studies report filament failures occurring at higher pressure drops at the nozzle exit [65,72,73].

**Figure 14:**
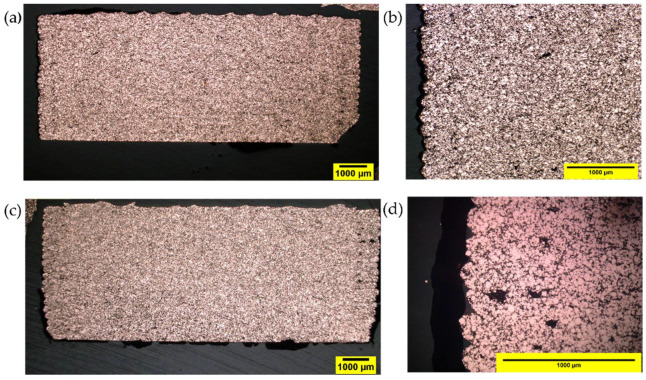
The optical microscopic image of a cross-section of MEX specimen piece F1_ST filaments: (**a**,**b**) Printing speed 10 mm s^−1^ (magnification of 20× and 200×); (**c**,**d**) printing speed 60 mm s^−1^ (magnification of 20× and 200×).

The micrograph of MEX-produced specimens based on F1_ST shows good adhesion among the adjacent deposited strands and a constant layer height (Figure 13a). The optical micrograph at higher magnifications (Figure 14b) reveals a good copper powder distribution in the entire cross-section of a printed specimen. Very few voids can be seen primarily at the corners of the examined section. The over extrusion appears to be highly efficient to reduce pores and compensate for the porosity inside the filaments (Figure 4). However, pores can be observed between the outer perimeter and the infill of the parts. These pores can be reduced by the overlapping of the perimeter and infill, as observed in previous studies [29]. The pores developed mostly in the last layers of the bending specimen as the voids are concentrated in the top part of the specimen surface. As the printing progresses and the layers are farther from the heated platform, the temperature of the bottom layer on which the new layer is deposited decreases [74]. A lower contact temperature means a lower mobility of the polymer chains and higher viscosity, and results in less bonding between layers. One possible way to reduce this porosity is to have a variable flow multiplier, which is offered in some slicer software. The effect of the part temperature in the bonding between layers can be also observed when comparing parts printed at different speeds, since higher porosity was observed at lower printing speed (10 mm s^−1^, Figure 14a) than at higher speed (60 mm s^−1^, Figure 14d). At higher printing speeds, the time between the deposition of one layer and the next is smaller, reducing the cooling of the deposited material and improving the bonding between layers. Nevertheless, the defects between the perimeter and infill of the parts are larger at higher (Figure 14c) than at lower (Figure 14b) speed, due to the perimeter defects at a higher printing speed (Figure 12).

**Figure 15:**
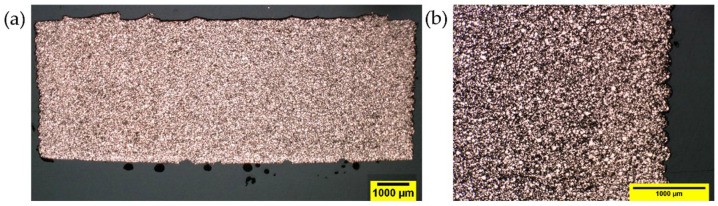
F2_ST MEX bending specimens produced at 60 mm s^−1^: With a magnification of (**a**) 20×; (**b**) 200×.

**Figure 16:**
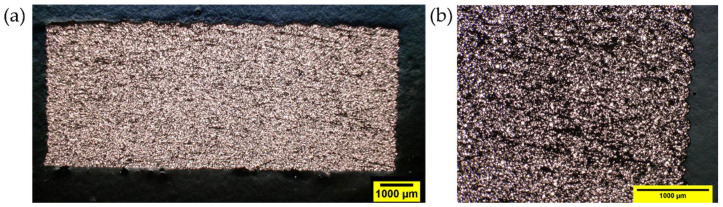
F3_T MEX bending specimens produced at 60 mm s^−1^: With magnification of (**a**) 20×; (**b**) 200×.

MEX-produced specimens based on F2_ST filaments also revealed a good copper powder distribution and few voids in the printed specimens (Figure 15). The cross-section of F2_ST specimens was similar to that of F1_ST specimens, but less perimeter-infill defects were observed for F2_ST (Figure 14b) than for F1_ST (Figure 14c) at 60 mm s^−1^ (For the cross-section of F2_ST specimens with 10 mm s^−1^, please refer to the supporting information Figure S2). The largest porosity was observed in the MEX-produced specimens based on F3_T (Figure 16). The large pores of the F3_T specimens are homogeneously distributed in the whole cross-section, which indicates a systematically poor feedstock extrusion. As previously described, the poor powder-binder interaction and uneven coating of the powder result in weak and soft F3_T filaments, which cannot be continuously extruded by the MEX system. Moreover, the poor powder-binder adhesion could result in separation at the printing nozzle, hindering the extrusion.

**Figure 17:**
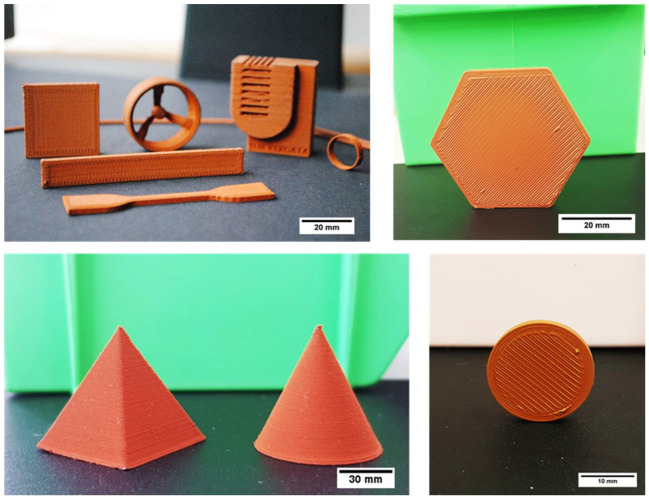
Parts of several shapes produced by MEX of copper-filled feedstocks (F1_ST filaments).

3D printed specimens were produced based on the best quality filament (F1_ST) with good printing quality and no visible defects (Figure 17) with the optimized printing parameters.

## 4. Conclusions

Three distinct multi-component binder systems were individually compounded with 55 vol.% copper to prepare three different feedstocks. Filaments were extruded from each feedstock, and their processability by MEX was investigated. All filaments showed good dispersion of the powder in the polymeric binder system in SEM analysis, but it was observed that binder adhesion to particles was different among the different feedstocks. A similar viscosity was achieved for all feedstocks by adjusting the temperature after knowing their thermal properties (i.e., melting point (T_m_) and crystallization temperature (T_crys_)). The mechanical properties were also measured to relate it to the printability of the filaments. It was observed that the feedstock with the simplest formulation (i.e., F3_T) had the lowest UTS and elongation at break since it had the lowest binder adhesion to the copper powder; therefore, it was susceptible to breakage at the extrusion head when printed at higher speeds. These results demonstrate once again that viscosity is usually not the limiting factor when determining the printability of highly filled filaments, but rather the mechanical properties of the filaments. The MEX optimization of the feedstocks was performed by depositing material at different speeds and volumetric flow (extrusion multiplier) and adjusting the printing temperature. It was observed that increasing the extrusion multiplier and temperature leads to improvements in the appearance and reduction of the porosity, but up to a certain level, above which it is counterproductive. For the feedstock materials that could be printed at two speeds, micrographs of a cross-section of printed parts revealed that as the printing speed increased, the porosity was reduced due to a better bonding between layers as the time between deposition of consecutive layers is smaller and the temperature is higher. Future work will compare the debinding and sintering behavior of the different feedstocks to demonstrate the influence of the binder on the properties of sintered specimens, which is the goal of preparing these different feedstocks.

## Figures and Tables

**Table 1:** Particle size data of copper powder from sieve analysis as declared by Carpenter Powder Products (Woonsocket, RI, USA).

Particle Size	Distribution
D10 (µm)	6.8
D50 (µm)	16.0
D90 (µm)	33.6

**Table 2:** Extrusion parameters of feedstocks of different composition and temperatures were selected based on the melting temperature of the binder components.

Feedstock	Metal Powder (55 vol.%)	Binder Systems	Compounding Temperatures (°C)	Die Temperatures (°C)	Torque (%)	Die Pressure (Bar)
F1_ST	Copper	B1_ST	120–200	200	-	-
F2_ST	Copper	B2_ST	30–170	170	28 ± 2	16 ± 3
F3_T	Copper	B3_T	30–170	170	27 ± 1	7 ± 2

**Table 3:** Processing parameters used during extrusion of binder and feedstock filaments; temperatures were selected based on the melting temperature of the binder components.

Filaments	Extrusion Temperatures (°C)	Die Temperature (°C)	Speed (rpm)
B1_ST	190–205	205	40
B2_ST	155–170	170	40
B3_T	155–170	175	35
F1_ ST	190–205	200	50
F2_ ST	155–160	160	37.3
F3_T	155–170	175	35.1

**Table 4:** Parameters varied during printing of final bending specimens (with a length of 80 mm, a width of 10 mm, and a thickness of 4 mm). Please notice that temperatures were selected based on the melting temperature of the binder components.

Filaments	Extrusion Temperatures (°C)	Printing Speed (mm s^−1^)	
F1_ST	255	10	60
F2_ST	200	10	60
F3_T	180	10	60

**Table 5:** The diameter and ovality of all the types of the feedstock filament spools used in the manufacturing of the MEX specimens: (**a**) F1_ST; (**b**) F2_ST; (**c**) F3_T.

Type of Feedstock	Diameter (mm)	Ovality (mm)
F1_ST	1.770 ± 0.017	0.011 ± 0.003
F2_ST	1.762 ± 0.013	0.013 ± 0.001
F3_T	1.769 ± 0.011	0.021 ± 0.001

**Table 6:** Crystallization and melting temperatures of binders and feedstocks.

Material	Crystallization Peaks		Melting Peaks	
	Low T_crys_ (°C)	High T_crys_ (°C)	Low T_m_ (°C)	High T_m_ (°C)
B1_ST	-	108.59 ± 0.93	-	153.34 ± 1.44
B2_ST	58.24 ± 0.57	115.48 ± 0.18	55.1 ± 0.21	123.67 ± 0.31
B3_T	-	90.51 ± 0.06	-	103.52 ± 0.04
F1_ST	-	119.11 ± 0	-	153.96 ± 0.24
F2_ST	57.55 ± 0.16	115.46 ± 0.09	53.79 ± 0.08	123.86 ± 0.24
F3_T	-	94.95 ± 0.07	-	104.29 ± 0.07

**Table 7:** Mechanical properties of the binder and feedstock filaments measured by tensile testing.

Material	UTS (MPa)	Strain at Break (%)
B1_ST	7.9 ± 1.4	546.6 ± 156.9
B2_ST	9.9 ± 0.14	16.6 ± 1.4
B3_T	7.6 ± 0.14	583.75 ± 78.4
F1_ST	7.95 ± 0.12	40.5 ± 4.3
F2_ST	9.8 ± 0.17	3.3 ± 1.16
F3_T	3.0 ± 0.12	1.5 ± 0.24

## Data Availability

Not applicable.

## Supplementary Materials

The following supporting information can be downloaded at: https://www.mdpi.com/article/10.3390/polym14224962/s1, Figure S1: The diameter of the filaments spool used in the manufacturing of the MEX specimens: (a) Feedstock F1_ST; (b) Feedstock F2_ST; (c) Feedstock F3_T; Figure S2: F2_ST MEX bending specimens produced at 10 mm s^−1^: With a magnification of (a) 20×; (b) 200×.
