# Supplementary material for: Influence of Binder Composition and Material Extrusion (MEX) Parameters on the 3D Printing of Highly Filled Copper Feedstocks
Source: Polymers (Basel). 2022 Nov 16;14(22):4962. doi: 10.3390/polym14224962 (PMC9692767; doi:10.3390/polym14224962)
Supplement: Supplementary file 1 [file polymers-14-04962-s001.zip › polymers-2021195-supplementary.pdf]

Influence of binder composition and material extrusion (MEX) parameters on the 3D printing of highly filled copper feedstocks

Mahrukh Sadaf [1,2]*, Santiago Cano [3]*, Joamin Gonzalez-Gutierrez [3,4]*, Mario Bragaglia [1], Stephan Schuschnigg [3], Christian Kukla [5], Clemens Holzer [3], Lilla Vály [6], Michael Kitzmantel [6] and Francesca Nanni [1]

1 University of Rome "Tor Vergata", Department of Enterprise Engineering "Mario Lucertini", and INSTM RU Roma-Tor Vergata, via del Politecnico 1, 00133 Rome, Italy
2 University of Ljubljana, Faculty for Mechanical Engineering, Laboratory of Experimental Mechanics, Ljubljana, Aškerčeva Ulica 6, 1000 Ljubljana, Slovenia
3 Montanuniversitaet Leoben, Institute of Polymer Processing, Otto Gloeckel-Straße 2, 8700 Leoben, Austria
4 Luxembourg Institute of Science and Technology, Material Research and Technology Department, Functional Polymers Research Unit, 5, rue Bommel, L-4940 Hautcharage, Luxembourg
5 Montanuniversitaet Leoben, Industrial Liaison Department, Peter-Tunnerstraße 27, 8700 Leoben, Austria
6 RHP-Technology GmbH, Austrian Research Center, 2444 Seibersdorf, Austria
* Correspondence: mahrukh.sadaf@fs.uni-lj.si (M.S.); santiago.cano@outlook.es (S.C.); joamin.gonzalez-gutierrez@list.lu (J.G.-G.)

Supplementary Materials

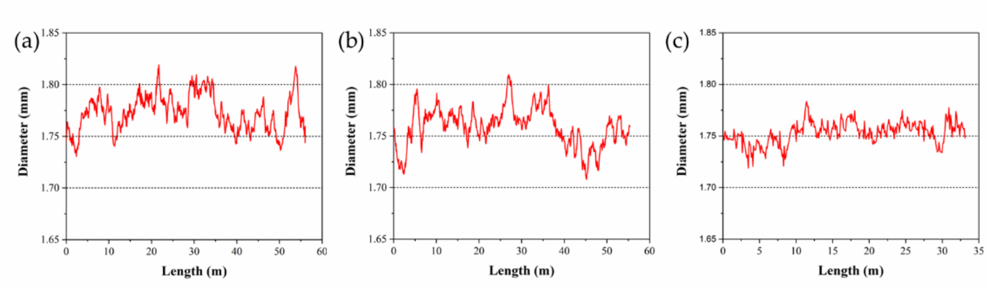

Figure S1. The diameter of the filaments spool used in the manufacturing of the MEX specimens: **(a)** Feedstock F1_ST; **(b)** Feedstock F2_ST; **(c)** Feedstock F3_T.

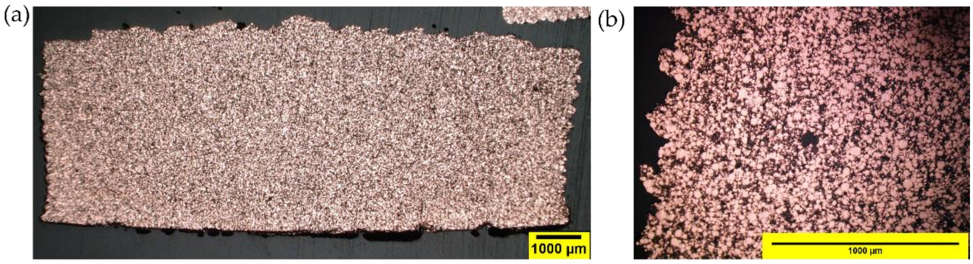

Figure S2. F2_ST MEX bending specimens produced at 10 mm s^{-1}: With a magnification of **(a)** 20x; **(b)** 200x.
